# Characterizing functional connectivity patterns during saliva swallows in different head positions

**DOI:** 10.1186/s12984-015-0049-x

**Published:** 2015-07-24

**Authors:** Iva Jestrović, James L. Coyle, Ervin Sejdić

**Affiliations:** Department of Electrical and Computer Engineering, Swanson School of Engineering, University of Pittsburgh, Pittsburgh, PA USA; Department of Communication Science and Disorders, School of Health and Rehabilitation Sciences, University of Pittsburgh, Pittsburgh, PA USA

**Keywords:** EEG, Graph theory, Brain network, Dysphagia, Swallowing, Chin-tuck

## Abstract

**Background:**

The anatomical rationale and efficacy of the chin tuck in improving airway protection for some people with swallowing disorders have been well researched and established. However, there are still open questions regarding whether brain activity for swallowing control is altered while performing this chin-tuck maneuver.

**Methods:**

In this study, we collected EEG signals from 55 healthy adults while swallowing in the neutral and chin-tuck head positions. The time-frequency based synchrony measure was used to form brain networks. We investigated both the small-world properties of these brain networks and differences among the constructed brain networks for the two head positions during swallowing tasks.

**Results:**

We showed that brain networks for swallowing in both head positions exhibit small-world properties. Furthermore, we showed that swallowing in the chin-tuck head position affects brain networks in the *Alpha* and *Gamma* frequency bands.

**Conclusions:**

According to these results, we can tell that the parameter of head position should be considered in future investigations which utilize EEG signals during swallowing activity.

## Introduction

Dysphagia refers to any kind of swallowing disorder, and it may occur for many different reasons. In the case of neurogenic dysphagia, swallowing difficulties occur due to lesions in disparate cortical and subcortical brain regions, or other structures of the central and peripheral nervous systems [1]. Some of the most common causes of oropharyngeal dysphagia are stroke [2], Parkinson’s diseases [3], cerebral palsy [4], and physical traumatic brain injuries [5]. Compromised airway protection and aspiration of food and liquids are the most significant immediate clinical result of dysphagia [6, 7]. Aspiration can lead to lethal outcomes such as airway obstruction, or develop into pneumonia which, according to previous studies, carries a mortality rate of 20 % to 50 % for dysphagia sufferers [8–10].

Swallowing in the chin-tuck position (head and neck flexion) is one of several postural techniques that allows some patients with specific swallowing abnormalities to swallow more safely [11, 12]. The chin-tuck maneuver involves flexing the head and neck approximately forty-five to sixty degrees, approximating the chin to the anterior upper chest while swallowing. This posture has been shown to produce several biomechanical advantages during swallowing for patients who have impaired ability to maintain posterior containment of food and liquids in the oral cavity until they are ready to swallow, or who have delayed onset of the pharyngeal stage of the swallow; both of which expose the unprotected airway to aspiration. When the head is in chin-tuck position, the vallecular cavities between the tongue base and epiglottis widen, which enables them to sequester material exiting the oral cavity before pharyngeal swallowing begins. This posture also repositions the posterior wall of the pharynx in closer proximity to the tongue base which narrows the pharynx, and subsequently, the inlet to the laryngeal vestibule, thereby reducing aspiration risk and increasing airway protection [12–15]. In the first study of its efficacy, swallowing in the chin-tuck head position eliminated aspiration in 50 % of patients who were aspirating liquids due to a delayed onset of the pharyngeal stage of swallowing [16], while the remaining 50 % of patients in this study did not benefit from the maneuver due to impairments for which the chin-tuck maneuver was disadvantageous. However, some studies indicate that swallowing in the chin-tuck head position is principally a compensatory maneuver that is effective in prolonging airway closure duration while volitionally deployed by the person swallowing, and that it did not only produced an effect that failed to persist after the posture was withdrawn [17]. On the other hand, studies showed that therapy and rehabilitation of patients with neurogenic dysphagia are highly correlated with the brain’s plasticity and ability to reorganize sensory and motor cortex after events such as stroke [18]. This neural reorganization produces compensatory changes in the swallowing sensory and motor subsystems [19, 20]. Therefore, efforts to better understand the potential central nervous system effects of therapeutic interventions used to restore swallow function might elucidate how maneuvers like the chin-down posture influence cerebral plasticity for swallowing after stroke and other neurological diseases.

Advanced techniques, such as functional magnetic resonance imaging (fMRI), positron emission tomography (PET), magnetoencephalography (MEG), and electroencephalography (EEG) provide significant insight into brain activity during swallowing. While fMRI and PET are characterized by very good spatial resolution, their shortcoming is that they fail to capture precise temporal information. MEG and EEG have better temporal performance; however, the limitation of MEG is that it cannot propagate a magnetic field into the areas that generate neural impulses in deeper layers of brain tissue. EEG demonstrates a degree of superiority when compared with other techniques. Besides having good temporal resolution, EEG records neural brain activation, which is considered a direct link to brain activity. Another advantage of EEG is that it is not sensitive to interactions or proximity to any material, and it also enables patients to remain in a natural body position during testing while fMRI requires the supine position which is a dangerous and unnatural position for dysphagic patients to assume while swallowing.

EEG also lends itself to analysis techniques which provide insight into the interactions between brain regions. It enables the measure and analysis of functional interactions between different brain regions by way of the graph theoretical approach. Graph theory mathematically describes the relationships between and among vertices (e.g., nodes). Interconnections between vertices are accomplished by edge connections. Most importantly, for functional interactions between different brain regions, graph theory provides an opportunity to determine the connective relationships between and among neighborhoods of vertices. Studies have shown that graph theory is suitable for analyzing functional connectivity in the human brain network and it has been widely used in different human and animal neuroscience studies because it facilitates easier analysis of the differences and similarities of brain networks [21–24]. Connections between various cerebral centers enable two-way communication between those centers and are important in the function of the brain as well as in its organizational development. Investigation of these communication networks in the brain could provide important insights into the core of human brain function. Network analysis has been previously used for interpreting other physiological signals [25]. These previous contributions lead us to the conclusion that it would be informative to apply the network analysis approach for the investigation of swallowing brain activities.

In a brain network, vertices refer to brain regions of interest, while edges are solely the functional connections between two brain regions. The position of the vertices and edges in a networks will define the topological structure of the brain network. Topological structure will determine if synchronization of neural activity between brain regions is organized more regularly or more randomly. Regular and random networks have different local clustering and interconnection path length characteristics. Regular networks are characterized by higher local clustering and longer path length between brain regions. This organization provides greater functional connectivity among the brain regions; however, communication efficiency is reduced between brain regions. Random networks have a lower local clustering and shorter path length between any two connected brain regions, which provides efficient communication between brain regions but poorer overall functional connectivity in the brain. Brain regions whose synchronization with one another is optimally organized have so-called “small-world” architecture topological properties. Small-world architecture topology of the networks share some characteristics with regular networks as well as some characteristics with random networks. Networks characterized by small-worldness have strong local clustering (i.e., a characteristic of regular networks) and a short path length connecting any two brain regions (i.e., a characteristic of random networks). Modeling studies have shown that neural networks with small-world properties are characterized by easier and more efficient communication between farther-apart neurons [26, 27], which correlates to optimally synchronized neural activity between different brain regions within a brain network. Investigation of small-worldness is of particular interest for analysis in dysphagia research because it could explain how neural reorganization occurs after cerebral injury, and lead researchers to interventions which would speed and optimize recovery from stroke and other neurological diseases. The small-word properties of constructed brain networks and the differences between these brain networks for two types of head positions will form the basis of our research.

Analysis of the brain functional connectivity during swallowing has been previously examined using fMRI time series. Luan et al. [28] showed that functional connectivity of the network obtained with fMRI during swallowing has small-world properties. Babaei et al. [29] showed greater functional connectivity in the area of the anterior and posterior insula. Humbert et al. [30] used several stimuli to investigate functional connectivity of the insular region. They showed greater clustering within posterior insula, and greater diversity of connections within the anterior insula. Even though fMRI provided significant insight into the brain functional connectivity during swallowing, to our knowledge, analysis of the functional connectivity of swallowing brain network have not been done using the data obtained with the EEG signals.

In this study, we investigated the small-world properties of brain networks for swallowing in two head positions: the neutral or natural position, and the chin-tuck head position, which is commonly used to alter swallowing biomechanics in certain types of dysphagia. We also seek to investigate whether there are differences in brain networks when individuals swallow using these two postures. While studies have given a physiological explanation of anatomical changes in the pharynx during the swallowing chin-tuck position, it remains unclear whether or how this maneuver affects brain activity. Investigating this brain activity during swallowing could explain if better control of swallowing in the chin-tuck position is simply due to the artificially modified configuration of the oropharyngeal mechanism caused by the change in posture or if is due to alterations in muscle activity caused by a transformation in cerebral function prompted by the postural change itself. Areas of brain damage induced by neurological pathologies that cause dysphagia may overlap with the brain regions involved in the control of swallowing in the chin-tuck position. Defining characteristics of the brain network and defining brain regions involved in these commonly deployed compensatory maneuvers could potentially explain if there is a central explanation for the limited efficiency of this technique for eliminating aspiration.

In addition, our investigation of small-world networks during swallowing may provide insight into the information transmission efficiency between groups of neurons involved in these two swallowing tasks, while increasing understanding of the differences in the small-world properties of brain networks constructed from swallowing in the neutral and chin-tuck head positions. We aim to determine if better small-world properties of the brain networks for swallowing in chin-tuck position provides a neurological explanation as to why the chin-tuck head position is an effective therapeutic technique for treating dysphagia. Therefore, we hypothesized that brain networks in both the neutral and the chin-tuck head positions have small-world properties, and that the brain network is different for swallowing in the neutral head position when compared with the brain network for swallowing in the chin-tuck head position.

## Methodology

### Data acquisition from participants

Data was collected from 55 healthy subjects, aged from 18 to 65, all of whom provided informed consent, as well as information about age, gender, height, and weight. The protocol was approved by the Institutional Review Board at the University of Pittsburgh.

**Fig. 1:**
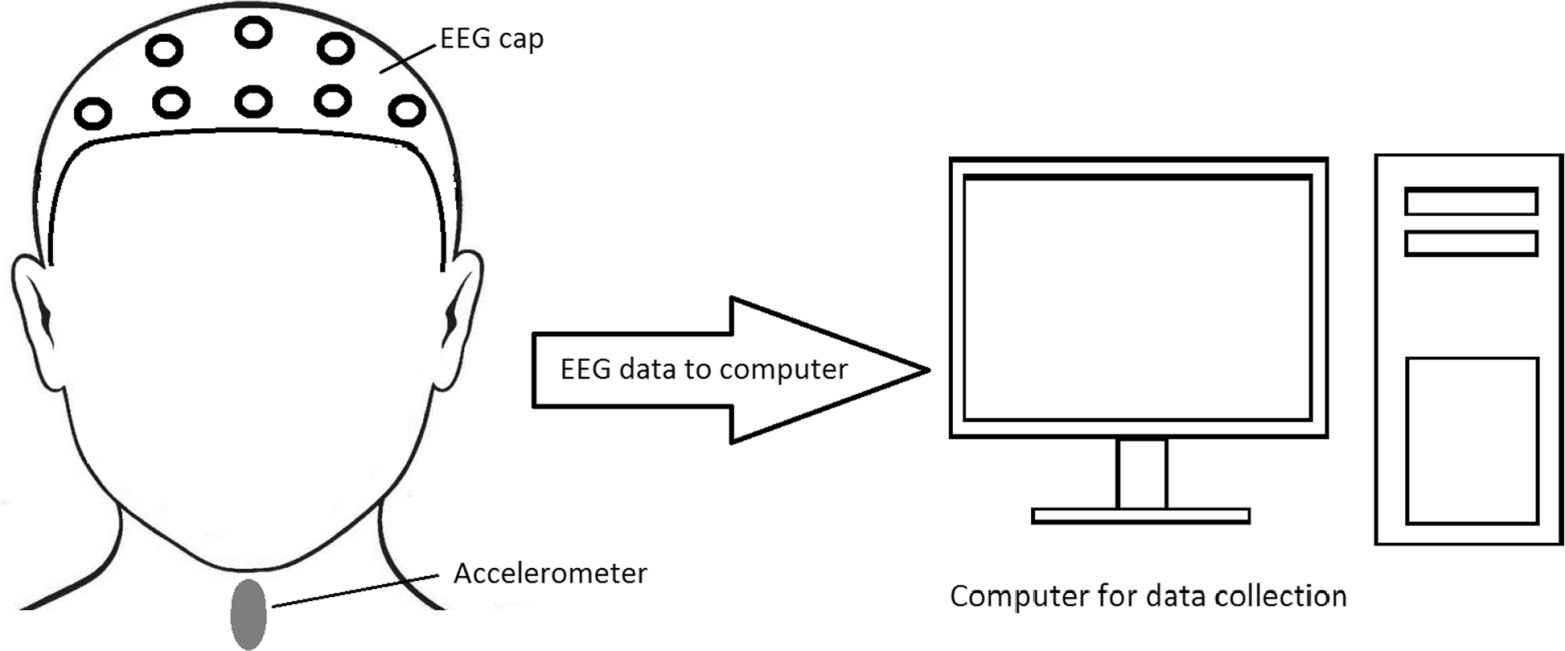
The experimental procedure used in this study

In this study, signals were collected from 64 EEG electrodes positioned according to the 10-20 international electrode system [31]. Electrode positioning was accomplished using the actiCAP active electrodes (BrainProducts, Germany), and signal amplification was performed using the actiCHamp amplifier (BrainProducts, Germany). The P1 electrode was chosen as the reference (i.e., EEG voltage potentials are referenced to P1). During all data collections, the electrodes’ impedance was below 15 k *Ω*. The PyCorder acquisition software provided a 10 kHz sampling frequency, and this software was also used for saving collected data on a computer hard drive. During EEG recording, a dual-axis accelerometer was used to record vibratory correlates of pharyngeal motor activity associated with individual swallows. It also provided temporal evidence regarding the beginning and end of each swallow event which was used to enable segmentation of the EEG signals into swallow-specific data sets. It was positioned on the anterior aspect of the neck at the level of the cricoid cartilage of each participant. The method for determining swallowing segments with the dual-axis accelerometer is described in detail in one of our previous studies [32]. After proper set-up of all EEG equipment was complete, participants were asked to perform ten saliva swallows in their self-selected time base between each swallow, first five saliva swallows in the neutral head position and then five saliva swallows in the chin-tuck head position (Fig. 1).

### Pre-processing steps

Collected data was pre-processed with the EEGlab MATLAB toolbox [33]. All signals were downsampled to 256 Hz, and then band-pass filtered from 0.1 Hz to 100 Hz with an elliptical infinite impulse response (IIR) filter. Next, in order to remove noise associated with the power supply, all signals were filtered with an elliptical IIR notch filter with cut-off frequencies at 58 Hz to 62 Hz. Individual swallows were identified according to the segmentation points provided by the accelerometer signal. Segmented swallows were then visually inspected for the presence of possible artifacts, which could be produced by electrode activation from any source other than cerebral activity (e.g., noisy nearby equipment and instruments, bodily movement, etc. that can produce undesirable artifacts in the EEG signal) [34]. All present artifacts were removed using the Independent Component Analysis (ICA) algorithm [35]. EEG data samples that contained an artifact that could not be removed by the ICA algorithm without significantly damaging the signal were excluded from the study. Less than 5 % of EEG data samples were excluded due to excessive artifacts.

### Network constructions

Pre-processed signals were filtered with an elliptic band-pass filter into the commonly used frequency bands: *Delta* (<4 *Hz*), *Theta* (4−7 *Hz*), *Alpha* (8−15 *Hz*), *Beta* (16−31 *Hz*), and *Gamma* (>32 *Hz*). The time-frequency based phase synchrony measure was used to compute connectivity matrices for each of the bands of interest.

#### Time-frequency based phase synchrony measure

After pre-processing and segmentation of the EEG signals, connectivity matrices were formed by estimating the synchrony between signal channels. There are several methods which have been previously used for calculating the synchronization between signals pairs such as: correlation, coherence, directed transfer function [36], partial directed coherence [37], Granger causality [38], etc. However, these methods are not suitable for dealing with non-stationary signals. In one of our previous studies, we showed that EEG signals during the swallowing activity are non-stationary [39]; therefore, for generating connectivity matrices, we used the time-frequency based phase synchrony measure method proposed by Aviyente et al. [40]. This method is called the reduced interference Rihaczek distribution.

Synchronization between any two signals can be estimated as the instantaneous phase of the signals around some desired frequency point. The signal in the time-frequency domain can be represented as:

(1) $$ X(t,\omega)=a(t)exp(j(\omega t + \phi(t))),  $$

where *a*(*t*) is the amplitude and *ϕ*(*t*) is the phase of the signal. From the vantage point of the time domain for some desired frequency, the organization of two signals, *x* and *y*, can be estimated by the difference in their phase:

(2) $$ \Phi_{xy}(t)=\lvert n\phi_{x}(t) - m\phi_{y}(t) \rvert,  $$

where *n* and *m* are ratios of the locking frequencies, and *ϕ*_*x*_ and *ϕ*_*y*_ are phases of the signals *x* and *y*, respectively.

In the event where we have a signal which can be presented as a sum of independent signals, such as, *s*(*t*)=*s*_1_(*t*)+*s*_2_(*t*), the Rihaczek time-frequency distribution is defined as:

(3) $$ \begin{aligned} C(t,\omega) &\,=\, \frac{1}{\sqrt{2\pi}}e^{-j\omega t} (s_{1}(t)S_{1}^{*}(\omega) +s_{2}(t)S_{2}^{*}(\omega) + s_{1}(t)S_{2}^{*}(\omega) \\&\quad+ s_{2}(t)S_{1}^{*}(\omega)), \end{aligned}  $$

where *S*(*ω*) is the Fourier transform of the signal. The last two terms in Eq. (3) are the cross-terms. The problem associated with cross terms is that they exist at the same time points as the original signal; furthermore, these cross terms occupy the same frequency bands which are occupied by the original signal. In order to remove the cross-terms, the Choi-Williams (CW) kernel function, which is defined as *ϕ*(*θ*,*τ*)=*exp*(−(*θτ*)^2^/*σ*), is applied to the Rihaczek distribution [41]. As a result, we obtained the reduced interference distribution of Rihaczek distribution which can be written as:

(4) $$ C(t,\omega)=\iint e^{(-{(\theta \tau)}^{2} /\sigma)} e^{(j(\theta \tau)/2)} A(\theta,\tau) e^{-j(\theta t + \tau \omega)} d\tau d\theta,  $$

where $A(\theta,\tau)=\int s(u + (\tau /2))s^{*}(u - (\tau /2)) e^{j \theta u} du$. The *A*(*θ*,*τ*) term is the ambiguity function of the original signal, and the *e*^(*j*(*θτ*)/2)^ term is the kernel corresponding to the Rihaczek distribution.

After defining the time-varying phase spectrum, the phase difference between two signals is computed as:

(5) $$ \Phi_{12}(t,\omega)=arg \left [\frac{C_{1}(t,\omega)C_{2}^{*}(t,\omega)}{\lvert C_{1}(t,\omega) \rvert \lvert C_{2}(t,\omega) \rvert} \right ].  $$

Calculated values for the phase difference between signal pairs are used for calculating the phase locking value (PLV). PLV is a measure of the phase difference between signal pairs, and it is defined as:

(6) $$ PLV(t,\omega)= \frac{1}{N} \left \lvert \sum_{k=1}^{N} exp(j \Phi_{12}^{k} (t,\omega)) \right\rvert,  $$

where N is the number of trials. *PLV* has values in the range from 0 to 1, inclusive. *PLV* values tend to be higher when the phase difference does not vary significantly between trials.

### Network measures

The formed weighted undirected connectivity matrices are typically converted into binary undirected matrices. This study considers the use of binary undirected networks for the purpose of calculating network measures. The constructed weighted undirected connectivity matrices are thresholded in order to form binary networks. For a binary undirected network, *a*_*ij*_ refers to the connection between node *i* and node *j*. If a connection exists between two nodes (i.e., node *i* and node *j*), then *a*_*ij*_=1, but if *a*_*ij*_=0, then there exists no connection between two nodes (i.e., node *i* and node *j*). When comparing network measures, only matrices containing the same number of edges can be considered. Because there is no single accepted method for choosing a threshold value, we chose to form binary networks by thresholding the weighted networks according to the percent of the density of connections (e.g., if a threshold is set to 10 %, then 10 % of the strongest connections in the connectivity matrix will be assigned a value of 1 while the remaining 90 % connections will be assigned a value of 0). In this study, binary networks were constructed by thresholding from 5 % (i.e., sparse connections) to 100 % (i.e., full connections) of connections using increments of 5 %. For each of the constructed networks, network measures were calculated and then compared to extract differences and similarities between swallowing in the neutral and chin-tuck head positions.

We used the Brain Connectivity Toolbox (BCT) [42] running in MATLAB to calculate each of the discussed network measures:

- The degree of the node, *D*_*i*_, is the number of the edges that node *i* has with the rest of the nodes in the graph. The degree parameter is one of the fundamental network parameters in graph theory. Degree distribution is the summation of the degrees of all nodes from the network while the mean degree is the average of all the degrees of all nodes in the network.
  (7) $$ D=\frac{1}{N}\sum_{\substack{i\in N}}D_{i},  $$
  where *N* is the number of nodes.
- The clustering coefficient of the *i*-th node is *C*_*i*_. The clustering coefficient is a measure which describes the ratio between the number of existing edges between the nearest neighbor of the node and the maximum number of possible edges [43]. For a binary network, the clustering coefficient is calculated as:
  (8) $$ C_{i}=\frac{2E_{i}}{D_{i}(D_{i}-1)},  $$
  where *E*_*i*_ is the number of existing edges between adjacent nodes to node *i*, and *D*_*i*_ is the degree of the *i*-th node. In the case of a random network, the clustering coefficient is relatively low; whereas a higher clustering coefficient is found in networks which contain more densely connected clusters [44]. The mean clustering coefficient is defined as:
  (9) $$ C=\frac{1}{N}\sum_{j=1}^{N} C_{j}.  $$
- The shortest path length, *L*_*i*_, is the minimum number of edges needed for one node to be connected to another node [45, 46]. The mean shortest path length is the average shortest length between all possible combinations of only two nodes in the graph. Mathematically, the mean shortest path length between two nodes is defined as:
  (10) $$ L_{i,j}=\frac{1}{N(N-1)}\sum_{\substack{i,j\in N,i\neq j}}d_{i,j},  $$
  where *d*_*i*,*j*_ the shortest path length between node *i* and node *j*. Furthermore, the characteristic path length is calculated as the average of all shortest path lengths:
  (11) $$ L=\frac{1}{N}\sum_{i \in N} L_{i}.  $$
- The local efficiency, *E*_*local*_, is defined as the mean of the efficiencies of the subgraphs which are formed from the neighborhoods of each node [47]. The local efficiency can be calculated as:
  (12) $$ E_{local}=\frac{1}{N}\sum_{\substack{i\in N}}E(G_{i}),  $$
  where *E*(*G*_*i*_) is the efficiency of the subgraph, *G*_*i*_.

The clustering coefficient, *C*, and the mean characteristic path length, *L*, are the two parameters used to describe small-worldness. A network is considered to have small-world properties if it is characterized by a high clustering coefficient and short characteristic path length. Networks with small-world properties will have high formations of clustered subnetworks [48]. Mathematically, small-world attributes should satisfy two conditions:

(13) $$ \gamma=\frac{C}{C_{random}}\gg 1,  $$

where *γ* is a normalized clustering coefficient, and

(14) $$ \lambda=\frac{L}{L_{random}}\approx 1,  $$

where *λ* is a normalized characteristic path length. *C*_*random*_ and *L*_*random*_ are the mean clustering coefficient and the mean characteristic path of the random network, respectively. In order to calculate *C*_*random*_ and *L*_*random*_, we generated 100 random networks using the Markov-chain algorithm [24, 49]. From each random network we calculated the clustering coefficient and the characteristic path length. *C*_*random*_ and *L*_*random*_ are the averaged clustering coefficient and the characteristic path length calculated from each random graph. Finally, small-worldness is the ratio between normalized clustering coefficient and normalized characteristic path length:

(15) $$ S=\frac{C/C_{random}}{L/L_{random}}.  $$

If this ratio is higher than one (*S*>1), it can be said that network possess small-world properties.

### Data analysis

To determine the categorical statistical differences between features in different head positions, the Wilcoxon rank-sum test was used [50].

## Results

We analyzed 252 swallows in the neutral head position, and 233 swallows in the chin-tuck head position. Results of the network measures are presented as a mean value ± standard deviation, as a function of percent of network connections.

**Fig. 2:**
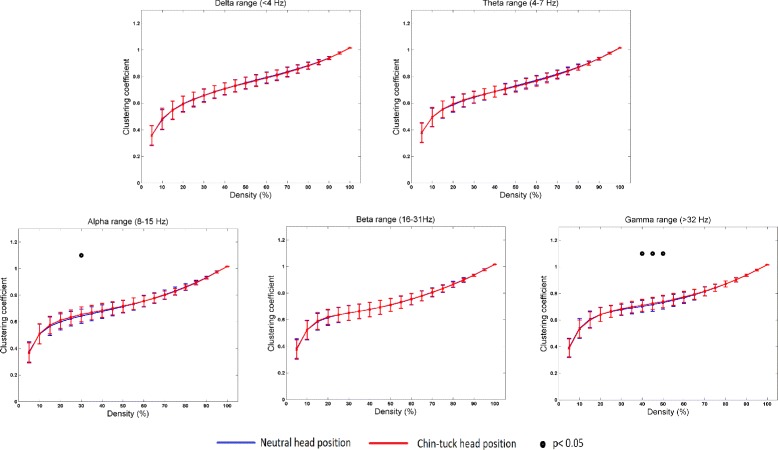
The value of mean clustering coefficient, *C*, for different threshold percentages and for different frequency bands

Figure 2 summarizes the mean value of the clustering coefficient for different connection densities (i.e., from 5 % to 100 %). The clustering coefficient did not exhibit statistically significant differences between different head positions for the *Delta*, *Theta*, and *Beta* frequency ranges (*p*>0.05). Swallowing in the chin-tuck head position showed a higher clustering coefficient for a 30 *%* connection density in the *Alpha* frequency range (*p*=0.02) when compared to swallowing in the neutral head position. Furthermore, swallowing in the chin-tuck head position showed a higher clustering coefficient for connection densities of 40 *%*,45 *%*, and 50 *%* in the *Gamma* frequency range (*p*<0.03) when compared with swallowing in the neutral head position.

**Fig. 3:**
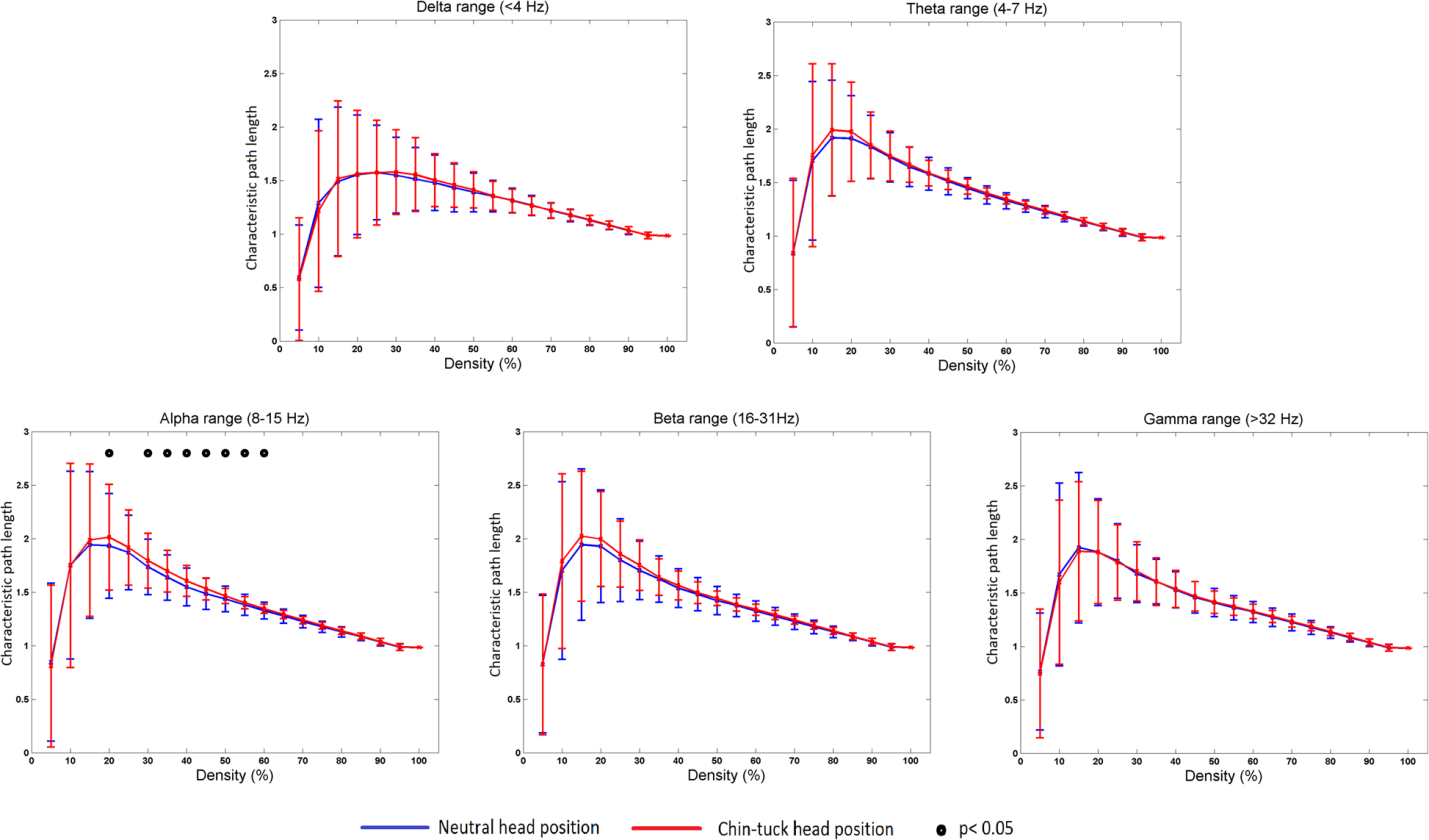
The value of mean characteristic path length, *L*, for different threshold percentages and for different frequency bands

Figure 3 summarizes the mean value of the characteristic path length for different connection densities. For connection densities of 20 *%*, 30 *%*, 35 *%*, 40 *%*, 45 *%*, 50 *%*, 55 *%*, and 60 *%* in the *Alpha* frequency range, swallowing in the chin-tuck position showed a higher mean value of the characteristic path length than swallowing in the neutral head position (*p*<0.05). In all other frequency bands (i.e., *Delta*, *Theta*, *Beta* and *Gamma* bands) there were no significant differences between different head positions (*p*>0.05).

**Fig. 4:**
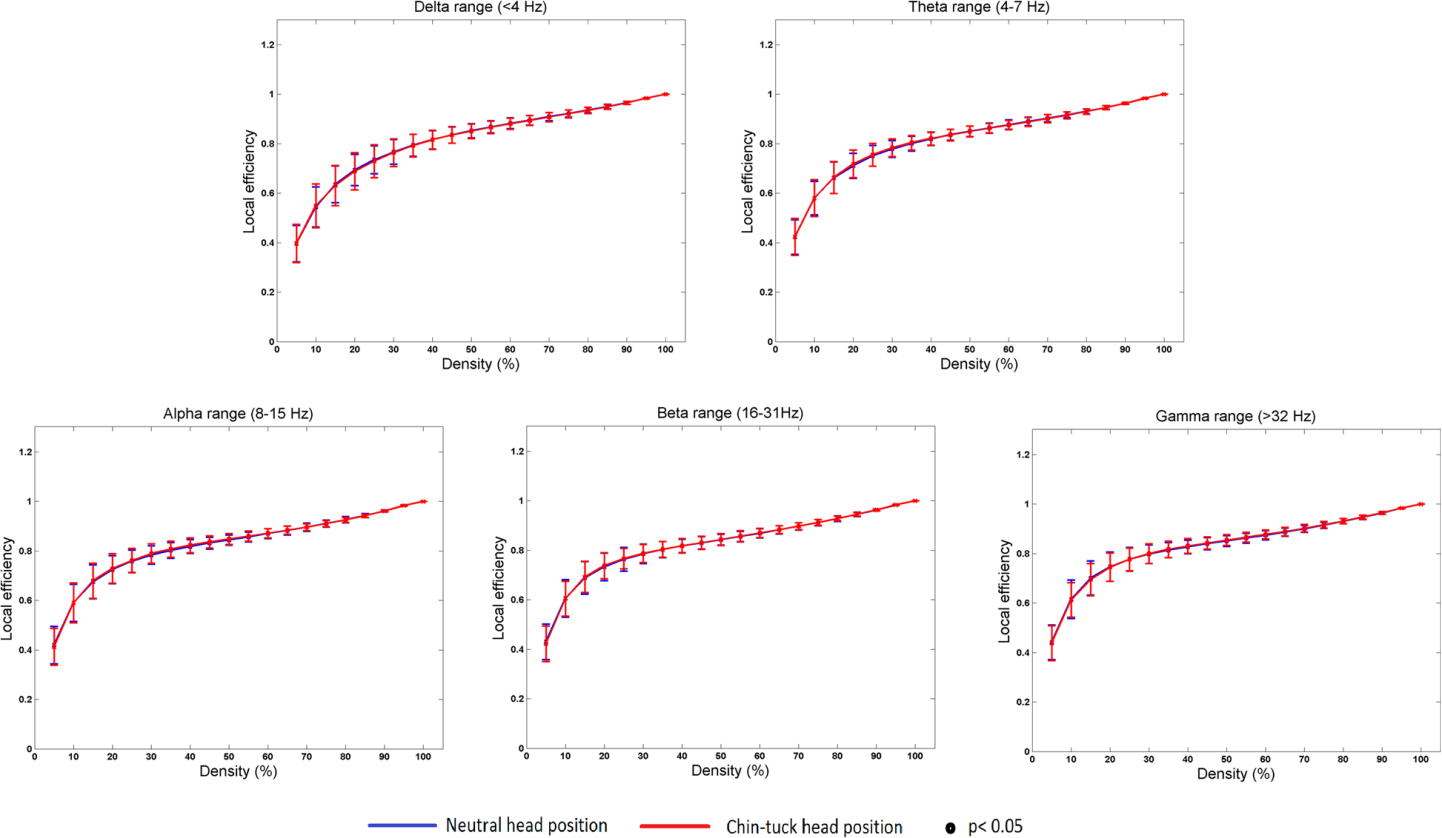
The value of local efficiency, *E*_*l*_, for different threshold percentages and for different frequency bands

**Fig. 5:**
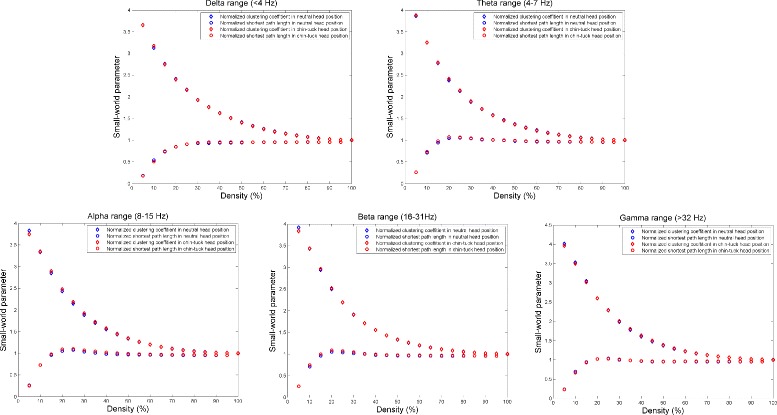
The value of mean normalized clustering coefficient *γ*, and the mean normalized characteristic path length, *λ*, for different threshold percentages and for different frequency bands

Figure 4 summarizes the mean value for the local efficiency, and Fig. 5 summarizes the mean normalized clustering coefficient (*C*/*C*_*random*_), and the mean normalized characteristic path length (*L*/*L*_*random*_) for different connection densities. None of these parameters showed statistically significant differences for the swallowing activity in different head positions. However, it should be noted from Figure 5 that the ratio between the normalized clustering coefficient and the normalized characteristic path length is greater than one in both head positions. Therefore, we can say that swallowing in both head position exhibited small-world properties of the network.

## Discussion

Our hypothesis that the constructed brain network for swallowing in the neutral and chin-tuck head positions has small-world properties was supported by our results. However, our hypothesis that the brain network is different for swallowing in the neutral head position compared with the brain network constructed for swallowing in the chin-tuck head position was partly supported for only some features (i.e., clustering coefficient and characteristic path length).

Our results did not show any statistical differences between swallowing in the neutral and swallowing in the chin-tuck head positions for the *Delta*, *Theta*, and *Beta* frequency bands. Previous studies reported that the *Delta* and *Theta* frequency bands activate during sensory stimulation [51, 52]. Also, it was reported that the *Beta* frequency band activates during anxious thinking and active concentration [53]. Therefore, we can say that there is no difference in activation of the sensory receptors between swallowing in the neutral and chin-tuck positions. Furthermore, there is no difference in the level of required cognitive concentration between swallowing in the neutral and chin-tuck positions with healthy patients.

Studies which previously investigated the origins of the EEG frequency bands suggested that the *Alpha* EEG band is associated with the inhibitory control required for efficient performance of cognitive and motor tasks [54–56], while the *Gamma* EEG frequency band is associated with the performance of cognitive and motor tasks [57–59]. Our results showed differences in the *Alpha* and *Gamma* EEG bands for both the clustering coefficient and the characteristic path length for the chin-tuck head position compared with the neutral head position. These results could be attributed to the higher cognitive demand and inhibition of the swallowing task in the chin-tuck head position. Even though the chin-tuck postural technique is considered a comfortable maneuver for patients, swallowing in the chin-tuck position is not natural. The unnatural position used when performing the chin-tuck technique results in changes in the pharyngeal dimensions [60]. These pharyngeal dimensional changes affect the attention and evoke the inhibition of the person who is performing swallowing in the chin-tuck position as well as potentially altering afferent and the resultant efferent signals emanating to and from the cerebral centers involved in processing the sensorimotor activities involved in each of the two conditions. While most aspects of normal swallowing in healthy people occur spontaneously (i.e., with no conscious effort), swallowing in the chin-tuck position demands additional cognitive contributions. Chin-tuck position swallowing recruits additional neural regions in the brain because this type of swallowing carries a higher cognitive demand and attention to a swallowing activity and a higher degree of inhibition, which explains the differences in our results.

A higher clustering coefficient for swallowing in the chin-tuck head position in the *Gamma* EEG frequency band could also be attributed to changes in muscle recruitment for this position. The *Gamma* EEG frequency band is well known to be modulated by muscular recruitment demands [57–59]. During swallowing in the chin-tuck position, the various regional muscles exhibit different pre-contraction lengths due to the altered configuration of the pharynx, which can produce a change in the resultant force of their contraction [15, 58]. This change leads to a possible explanation of the differences that we found in the *Gamma* EEG band between these two head positions. This means that decreased muscular recruitment in the chin-tuck head position correlates to greater functional connectivity in the brain, as evidenced by a higher clustering coefficient for the *Gamma* EEG frequency band, when compared with the neutral head position. That is to say, changes in posture that reduce muscular control for a swallowing task may increase the central functional motor control for the swallowing task in the brain.

A shortcoming of this study is that participants were instructed to perform five consecutive swallows in quick succession, which limiting the amount of saliva that can accumulate between swallows. It is possible that saliva-bolus volumes differed as a result of the allotted time for re-accumulation of saliva during the data collections for swallowing tasks. Also, we did not counterbalance the order of the two experimental positions, the chin-tuck and control (i.e. neutral posture) positions, when participants were swallowing. In order to overcome these limitations, future studies should investigate swallowing activities with other fluids using a specific bolus volume and consistency (e.g., water, nectar-thick, and honey-thick juice), or sufficiently allow saliva to re-accumulate to a specific range of volume, as well as imposing a random or counterbalanced order of presentation of the conditions. Also, it has been reported that swallowing saliva involves more cortical activity across the age-span and in disease than swallowing water or barium [61, 62]. Therefore, this issue should be considered in future investigations. Furthermore, a future study could also investigate differences in the brain networks constructed for each of the consecutive swallows.

Graph theory is a powerful technique which enables thorough analysis of brain functional connectivity and provides deep insight into brain activity. Analysis of brain function is obtained by mathematical calculation of the network measures that describe the architectural properties of the network. Besides the network measures that were used in this study (i.e., node degree, clustering coefficient, the shortest path length, and local efficiency), there are a number of other features (e.g., transitivity, modularity, network motifs, network graphlets, etc.) which could provide more distinctive information about a network’s architecture. Therefore, future swallowing studies should also consider network analysis using other features.

## Conclusion

In this study we investigated the differences between constructed brain networks corresponding to saliva swallows in both the neutral and chin-tuck head positions. Swallowing EEG signals were collected from 55 healthy adults, each of whom performed five saliva swallows in both of these head positions. We demonstrated that the constructed brain networks corresponding to these two tasks have small-world properties, which indicates that swallowing in either head position has optimal architectural organization and provides efficient communication between different brain regions for their respective task demands, and that neither disadvantageously disrupts connectivity between and among brain regions in healthy people. Clinically, we have observed patients with various neurological conditions fail to benefit from the chin-down posture despite being good candidates for its use on the basis of their swallowing impairment. It would be worthwhile to investigate this question in pathological states to elucidate the clinical importance of these findings in dysphagic patients and to determine whether the chin-down posture indeed maintains optimal architectural organization and regional connectivity among brain regions.

We also demonstrated that there exists a difference between swallowing in the neutral and chin-tuck positions within the *Alpha* and *Gamma* frequency bands. This difference implies that swallowing in the chin-tuck head position may modify central processing of swallowing sensorimotor circuits by either changing direct motor output, or through the influence of cognitive activity required for posturing. As a result, the cognitive impacts of the participant’s head position should be considered in future investigations concerning swallowing. Again, further investigation of this hypothesis in pathological states is warranted.
